# Pharmacological Therapeutics Targeting RNA-Dependent RNA Polymerase, Proteinase and Spike Protein: From Mechanistic Studies to Clinical Trials for COVID-19

**DOI:** 10.3390/jcm9041131

**Published:** 2020-04-15

**Authors:** Jiansheng Huang, Wenliang Song, Hui Huang, Quancai Sun

**Affiliations:** 1 Department of Medicine, Vanderbilt University Medical Center, 318 Preston Research Building, 2200 Pierce Avenue, Nashville, TN 37232, USA; 2 Center of Structural Biology, Vanderbilt University, 2200 Pierce Avenue, Nashville, TN 37232, USA; 3 School of Food and Biological Engineering, Jiangsu University, Zhenjiang 212013, Jiangsu, China

**Keywords:** RNA-dependent RNA polymerase, remdesivir, chloroquine, SARS-CoV-2, COVID-19, spike glycoproteins

## Abstract

An outbreak of novel coronavirus-related pneumonia COVID-19, that was identified in December 2019, has expanded rapidly, with cases now confirmed in more than 211 countries or areas. This constant transmission of a novel coronavirus and its ability to spread from human to human have prompted scientists to develop new approaches for treatment of COVID-19. A recent study has shown that remdesivir and chloroquine effectively inhibit the replication and infection of severe acute respiratory syndrome coronavirus-2 (SARS-CoV-2, 2019-nCov) in vitro. In the United States, one case of COVID-19 was successfully treated with compassionate use of remdesivir in January of 2020. In addition, a clinically proven protease inhibitor, camostat mesylate, has been demonstrated to inhibit Calu-3 infection with SARS-CoV-2 and prevent SARS-2-spike protein (S protein)-mediated entry into primary human lung cells. Here, we systemically discuss the pharmacological therapeutics targeting RNA-dependent RNA polymerase (RdRp), proteinase and S protein for treatment of SARS-CoV-2 infection. This review should shed light on the fundamental rationale behind inhibition of SARS-CoV-2 enzymes RdRp as new therapeutic approaches for management of patients with COVID-19. In addition, we will discuss the viability and challenges in targeting RdRp and proteinase, and application of natural product quinoline and its analog chloroquine for treatment of coronavirus infection. Finally, determining the structural-functional relationships of the S protein of SARS-CoV-2 will provide new insights into inhibition of interactions between S protein and angiotensin-converting enzyme 2 (ACE2) and enable us to develop novel therapeutic approaches for novel coronavirus SARS-CoV-2.

## 1. Introduction

Since its discovery in December 2019, the novel coronavirus-related pneumonia COVID-19 has continued to disseminate, with the current case count close to 1,214,466 cases, and more than 67,767 deaths according to the World Health Organization (WHO) as of 7 April 2020 [1,2]. Epidemiological studies suggest that the incubation period was estimated to be 1–14 days, whereas the serial interval was estimated to be 4–8 days. It takes about 3–7 days for the epidemic to double in the number of infections [3]. In addition, recent study demonstrated that there was about 5% of severe acute respiratory syndrome coronavirus-2 (SARS-CoV-2) among other patients with mild influenza-like symptom without risk factors [4]. These patients had only mild or moderate symptoms, so they are still active in the community during infection, which promotes the possibility of constant transmission. To have a better understanding of respiratory infectious disease transmission for pathogenesis and epidemiological spread of disease, a model for respiratory emissions was established and it was found that droplets containing the virus can be as small as 1 micron and a multiphase turbulent gas cloud from a human sneeze exhibited the property to travel great distance (7–8 m) [5]. This suggests that the gas cloud with its pathogen payload can span a certain space in a few seconds [5]. Giving a high rate of community spread, there is a need to change the public health policy from containment to mitigation of transmission, and determine the extent to which mild disease is contagious in the community, particularly among less vulnerable young adults for acquisition of SARS-CoV-2 infection [4]. This study also stresses the importance of close cooperation between clinicians, pharmaceutical companies and public health authorities [6]. Increase of clinical knowledge sharing will facilitate the rapid diagnosis and development of pharmacological approaches for treatment of SARS-CoV-2 infection [7,8]. The constant and rapid spread of novel coronavirus SARS-CoV-2 and its ability to disseminate from human to human has prompted scientists to develop new approaches for treatment of the novel coronavirus-related pneumonia COVID-19.

## 2. Coronavirus

Respiratory viral infection is a global health concern because the virus is contagious and may cause life-threatening respiratory infection and severe pneumonia in humans [9]. Currently, there are three single strand RNA (ssRNA) beta-coronavirus that have been identified, including severe acute respiratory syndrome (SARS) virus, Middle East respiratory syndrome (MERS) virus and SARS-CoV-2 [9]. Full-length genome sequence has identified that the genome sequences of SARS-CoV-2 obtained from five patients at the early stage of the outbreak were almost identical to each other and exhibited about 79.5% sequence identify to SARS-CoV [10,11]. Furthermore, it is found that SARS-CoV-2 is 96% identical at the whole-genome level to a bat coronavirus, which indicates that bats might be the intermediate host of this virus [12].There are several symptoms of coronavirus infection, such as sore throat, running nose, cough, sneezing, fever, viral conjunctivitis, loss of smell and taste and severe pneumonia [7,9,13,14,15,16]. It is also very challenging to make an accurate and timely diagnosis and it is not easy to distinguish when diagnosing between coronavirus and the influenza respiratory syndromes without RT-PCR diagnosis assay [17,18,19]. The number of deaths from COVID-19 is already more than the number of lives lost to SARS. There is a high rate of infection resulting from viral pneumonia, consequent inflammation and acute respiratory distress syndrome (ARDS) caused by SARS-CoV-2 [8,12]. Severe pneumonia, secondary infections and cardiovascular events were the major reason for death [13,20]. The pathological features of one case of death from severe infection with SARS-CoV-2 was investigated, and it was found that the pathological features of COVID-19 substantially resembled characteristics observed in SARS and Middle Eastern respiratory syndrome (MERS) coronavirus infection [21,22,23]. Recent study has shown that remdesivir and chloroquine efficiently suppressed the replication of SARS-CoV-2 in vitro [24]. One case of COVID-19 was treated with remdesivir in the United States [25]. After successful treatment, there was no detectable nucleic acid of SARS-CoV-2 from serum and oropharyngeal swab specimens and there was no gene mutation after comparing it with the previously reported genome sequence of SARS-CoV-2 [10,25]. This will be useful in understanding the pathogenesis of COVID-19 and discovering new therapies and clinical strategies against the infection. 

## 3. Potential Mechanisms of Coronavirus Invasion

### 3.1. Molecular Mechanisms of Coronavirus Invasion

**Figure 1:**
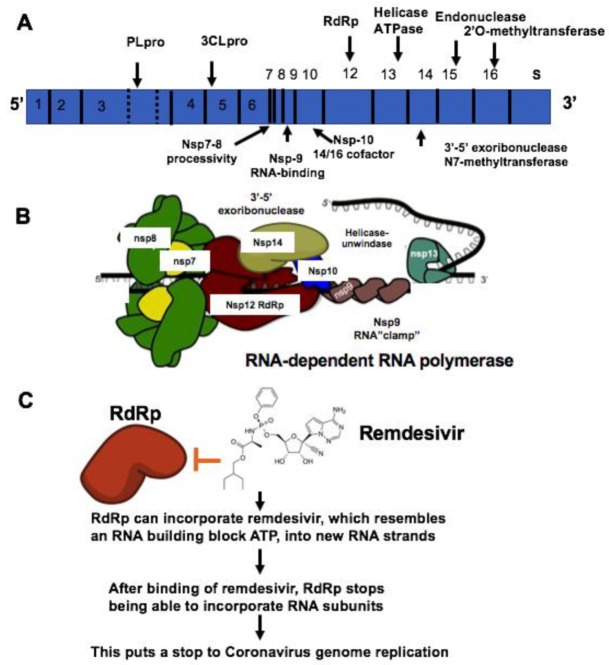
Mechanisms of remdesivir to inhibit RNA-dependent RNA polymerase (RdRp). (**A**) The genome composition model of single strand RNA (ssRNA) of coronavirus. (**B**) The RNA-dependent RNA polymerase RdRp mediated RNA replication during coronavirus infection. (**C**) Remdesivir functions as the ATP analog to inhibit RdRp.

The coronavirus (CoV) family has a large homogeneous “spike protein”. This spike protein (S protein) is responsible for interacting with the host cells, such as the pulmonary and parabronchial epithelial cell, and helps the coronavirus get through the epithelial cell membrane [26]. In addition, the alveolar epithelial cells have abundant expression of angiotensin-converting enzyme 2 (ACE2), which is targeted by the virus. The recognition of ACE2 by the S protein of the virus enables the invasion of the coronavirus into the human circulation system [27]. Recent study demonstrates that ACE2 is the SARS-CoV-2 receptor, which is required for cell entry [28]. Single-strand RNA (ssRNA) viruses such as the coronavirus family replicate the virus genomes by taking advantage of host cells. For example, after coronavirus approaches the ribosome of the epithelial cells or other host cells, it uses the ribosome of the host cell to replicate polyproteins. The replication and subsequent processes of precursor polyproteins can occur in the epithelial cells [29]. After the coronavirus’ polyproteins are expressed, two enzymes—specifically, coronavirus main proteinase (3CLpro) and the papain-like protease (PLpro)—are thought to be involved in cleaving the polyproteins into smaller products used for replicating new viruses [30]. In order to generate the daughter RNA genome, the coronavirus expresses an RNA-dependent RNA polymerase (RdRp), which is a crucial replicase that catalyzes the synthesis of a complementary RNA strand using the virus RNA template as shown in Figure 1 [31]. 

### 3.2. Factors Involved in Transcription and Release of Coronavirus Particles

Although genome replication and transcription are well known to be regulated by the viral RdRp, several host factors have been implicated in this process. RNA chaperones are usually nonspecific nucleic acid binding proteins, which have long disordered structures that promote RNA molecules to adjust conformational changes. For example, coronavirus nucleoproteins (N protein) have RNA chaperone activity and function as an RNA chaperone, which could help template switching [32,33,34]. In addition, recent studies demonstrate that glycogen synthase kinase 3 (GSK3) phosphorylates the N protein of SARS-CoV and further inhibition of GSK3 can effectively inhibit viral replication in Vero E6 cells infected with SARS-CoV [35]. Furthermore, heterogeneous nuclear ribonucleoprotein A1 (hnRNP A1) is involved in the pre-mRNA splicing in the nucleus and translation regulation in the host cells. Importantly, it has been shown that the nucleocapsid protein of SARS-CoV had binding ability to human hnRNP A1 with high affinity by using kinetic analyses with a surface plasmon resonance (SPR) approach. These studies suggest that hnRNPA1 is able to bind to SARS-CoV N protein to form a replication/transcription complex and control viral RNA synthesis [36].

In addition, several virus proteins and host factors are essential for the assembly and release of coronavirus. Homotypic interaction of M protein serves as the scaffold for the virus assembly and morphogenesis in the infected cells, specifically, both the interaction between the membrane (M) and S protein and the interaction between M and N protein promote the recruitment of structural components to the assembly location of host cells [37,38]. For example, both envelope (E) protein and N proteins are required to be co-expressed with M protein for the formation and release of virus-like particles (VLPs) after transfection of Vero E6 cells. Two crucial structural proteins, the M protein and E protein, play important roles in the coronavirus assembly. In addition, the E protein is involved in particle assembly by binding with M and further inducing membrane curvature [39]. Subsequently, coronavirus particles can be budded into the ER-Golgi intermediate compartment (ERGIC) of host cells, then trafficked in a smooth-wall vesicle and transported through the secretory pathway for assembly and release by exocytosis [40,41].

## 4. Current Treatment of Coronavirus

**Figure 2:**
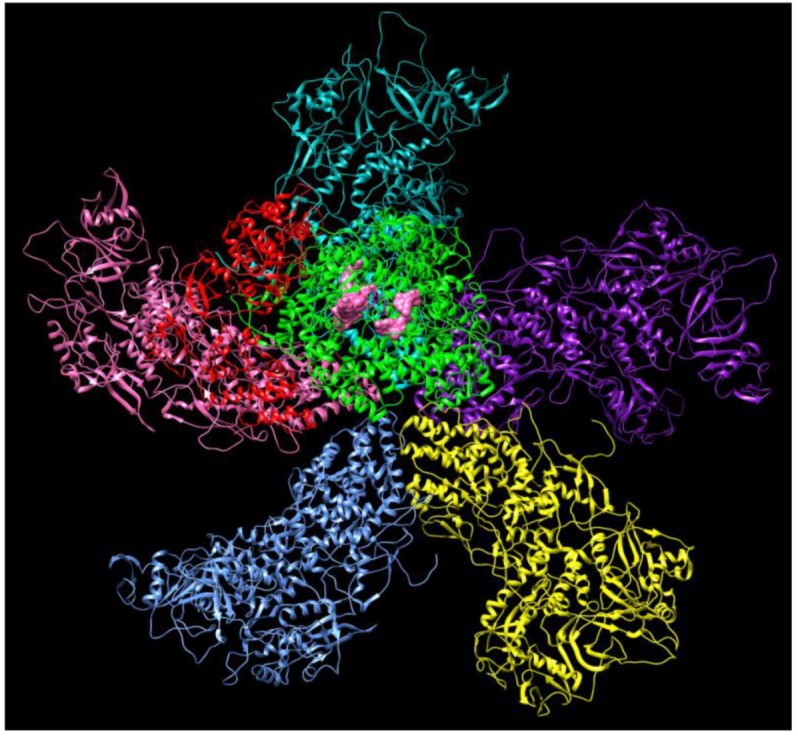
The structure of the RNA-dependent RNA polymerase (RdRp) complexes in the dinucleotide primed state a dsRNA virus (PDB: 6K32 RdRp complex). Chain A is shown as green, chain B is shown as red, chain C is highlighted in cyan, chain D is highlighted in pink, chain E is highlighted in blue, chain F is highlighted in yellow, chain G is highlighted in purple, chain P is highlighted in magenta and chain T is highlighted in orange. The structures demonstrate the interaction between the nucleotide substrates shown in pink and the conserved residues during the RdRp initiation, and the coordinated conformational changes preceding the elongation stage during replication.

There are two subunits of S protein, including the S1 subunit with a receptor-binding domain that engages with the host cell receptor ACE2, and the S2 subunit involved in regulating fusion between the viral and host cell membranes [42]. The S protein plays important roles in the induction of neutralizing-antibody and leads to T cell responses, so it is involved in protective immunity during infection with SARS-CoV [42]. Vaccines can be developed to specifically recognize the spike protein for SARS and ACE2 receptor [42]. However, mutations of the virus gene and the antibody-dependent enhancement (ADE) effect might affect the efficacy of previously developed biological vaccines, or even spur a counterproductive immune response, although spike protein sequences in SARS-CoV-2 and SARS exhibit some overlap [43,44,45]. Although it is of importance to develop vaccines and biological therapeutics to prevent the expansion of the SARS-CoV-2, a careful evaluation of possible immune complications is required before applying the vaccine to the public. Therefore, it may take several months or a few years to generate effective vaccines to prevent outbreak. Recent study demonstrates that SARS-CoV-2 has very similar genome sequence identity with severe acute respiratory syndrome-related coronavirus (SARS-CoV) and there is more than 90% sequence similarity in several essential enzymes, such as RNA-dependent RNA polymerase, papain-like proteinase (PLpro), 3CL-protease (3CL-pro) and spike glycoproteins [31]. Dissecting the structure of RdRp may provide new insights into the mechanisms of RNA replication as shown in Figure 2 [23]. Several drug candidates including ribavirin, lopinavir-ritonavir, and favipiravir, have been used previously to treat SARS or MERS, and these compounds may have potential in treating patients with SARS-CoV-2 from this current outbreak [31]. 

## 5. Current Diagnosis and Treatment of COVID-19

### 5.1. Current Molecular Diagnostic Assays

Researchers have posted the genome sequence information of SARS-CoV-2 isolated from pneumonia patients on the USCS Genome Browser [10,46]. This helps scientists to establish a real-time reverse transcription PCR (real time RT-PCR) diagnostic assay [47]. It is of importance to detect nucleic acids of SARS-CoV-2 in clinical diagnostics and biotechnology. Clustered regularly interspaced short palindromic repeats (CRISPR) technology, a simple and powerful tool, was initially developed to edit the genome of mammalian cells. Development of rapid, low-cost, and sensitive RNA detection may enhance point-of-care virus detection, genotyping, and disease progression monitoring. The RNA-targeting clustered regularly interspaced short palindromic repeats (CRISPR) effector Cas13a/C2c2 displays an unintentional effect of promiscuous ribonuclease activity along with specific RNA gene target recognition. Recently, a CRISPR-based diagnostic assay, that makes use of nucleic acid pre-amplification and CRISPR–Cas enzymology targeting either spike gene or Orf1ab gene, was established to specifically recognize desired RNA sequences [48]. Effective CRISPR guide RNAs (gRNA) and isothermal amplification primers can be designed to specifically target spike gene and Orf1ab gene. In the specific high-sensitivity enzymatic reporter unlocking (SHERLOCK) assays, coronavirus RNA can be amplified by using recombinase-mediated polymerase amplification with isothermal primers to boost the sensitivity at 37–42 °C. Subsequent Cas13a-mediated recognition of nucleic acid of coronavirus then cleaves the fluorescent RNA probe to separate a fluorophore from its quencher after Cas13a/C2c2 finds its target coronavirus RNA [49]. The cleaved fluorescent products can be readily detected and measured. This rapid CRISPR diagnostic assay can provide specific results in 1 h and will provide timely virus RNA detection with super-high sensitivity and the ability of single-base pair mismatch [48]. Therefore, development of rapid and robust diagnostic assay to measure the nucleic acid of SARS-CoV-2 is crucial for the accurate diagnosis of moderate and severe patients and screening of asymptomatic patients, especially under the current serious health care situation.

### 5.2. Treatment of COVID-19 with Remdesivir

**Figure 3:**
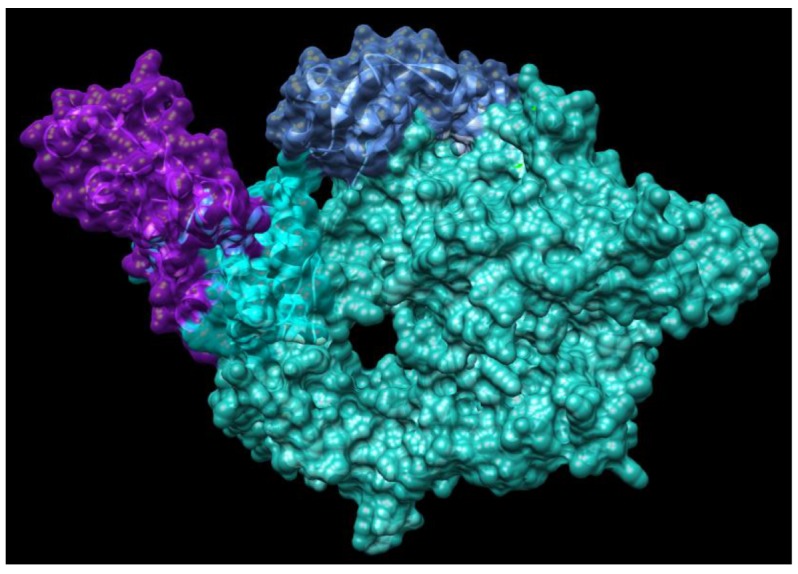
Structure of SARS-Coronavirus RNA polymerase NSP12 complex with NSP7 and NSP8 co-factors (PDB: 6NUR). Chain A is highlighted in light sea green, chain B is shown as blue, chain C is highlighted in cyan, chain D is highlighted in purple.

**Figure 4:**
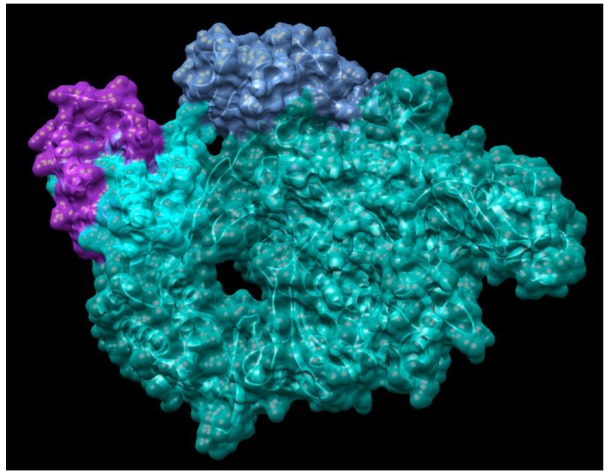
Structure of SARS-CoV-2 RNA polymerase NSP12 complex with NSP7 and NSP8 co-factors (PDB: 6M71). Chain A is highlighted in light sea green, chain B is shown as blue, chain C is highlighted in cyan, chain D is highlighted in purple.

Currently, there is no effective drug for treating COVID-19 although there was one case reported as having been treated successfully with compassionate use of remdsivir in the US. Recent studies have found that small molecules of remdesivir and chloroquine effectively suppress the replication of SARS-CoV-2 in vitro [24]. According to comparison of genome sequences of SARS-Cov-2 with SARS sequence, the catalytic domains of enzymes such as RdRp are highly conserved in these coronaviruses as shown in Figure 3 and Figure 4. More importantly, it is predictable that the protein sequence of the drug binding pocket of the enzymes is highly conserved [56]. Therefore, these enzymes and spike protein could be very promising drug targets for developing a therapeutic approach for COVID-19 as shown in Table 1 [57,58]. RdRp, also known as nsp12, which catalyzes the synthesis of coronavirus RNA, is an essential enzyme of the coronaviral replication/transcription machinery complex. Recent study revealed the structure of SARS-CoV-2 full-length nsp12 in complex with cofactors nsp7 and nsp8 using cryo-EM [59]. Excepting the conserved features of the polymerase component of the viral polymerase family and key domains for the coronavirus replication shown in RdRp, SARS-CoV-2 nsp12 has a newly featured β-hairpin domain at the N-terminal (PDB: 6M71) as shown in Figure 4. Further comparative analysis has shown how remdesivir binds to the binding pocket of RdRp of SARS-CoV-2 [59]. This structure provides new insight into the key enzyme of the coronaviral replication/transcription complex and lays a solid foundation for the design of new antiviral therapeutics targeting RdRp of SARS-CoV-2. 

Remdesivir (GS-5734, Gilead) was initially developed to examine its effect on inhibition of Ebola virus (EBOV) replication [60]. RdRp can incorporate remdesivir, which resembles an RNA building block ATP, into new RNA strands. After binding of remdesivir, RdRp stops being able to incorporate RNA subunits. This puts a stop to the coronavirus genome replication. Enzyme kinetics demonstrated that EBOV RdRp incorporated ATP and remdesivir-TP with comparable efficiencies. The selectivity of ATP for EBOV RdRp is four times against remdesivir-TP. In comparison, purified human mitochondrial RNA polymerase (h-mtRNAP) effectively distinguished against remdesivir-TP with a selectivity value of about 500-fold [60]. 

Remdesivir has been recently developed as a potential antiviral drug candidate against a wide array of RNA virus such as SARS-CoV and MERS-CoV5 infections in both in vitro cell experiment and preclinical studies with mice and nonhuman primate (NHP) models [61]. Recent study examined the efficacy of the broad-acting antiviral remdesivir in the rhesus macaque model of MERS-CoV infection. Interestingly, it was found that administration of remdesivir initiated 24 h prior to inoculation completely restrained MERS-CoV−caused respiratory disease, remarkably suppressed MERS-CoV virus replication in the respiratory system and abolished the progression of lung lesions [62]. These results demonstrated that remdesivir was a potential antiviral therapeutic against MERS and its efficacy could be further examined in clinical trials. Subsequently, recent study has tested the efficacy of remdesivir on inhibition of SARS-CoV-2 replication in vitro [24]. Vero E6 cells were infected with SARS-CoV-2. Different doses of the indicated antivirals were added to treat cells for 48 h. The viral yield from the cell supernatant was then detected by qRT-PCR. It is worth noting that two compounds, remdesivir significantly abolished virus infection at high affinity [24]. This also suggests the possibility that remdesivir has efficacy for related coronaviruses such as the novel coronavirus SARS-CoV-2 [62]. More importantly, administration to patients with COVID-19 of remdesivir has been shown to be effective in treating one patient for the purpose of compassionate use in the US, and there was no adverse event observed in association with infusion of remdesivir. 

### 5.3. Pharmacological Therapeutics Targeting Proteinase of SARS-Cov-2 

Due to the COVID-19 pandemic and global health concern, it is urgent to develop effective broad-spectrum virus replication inhibitors to manage patients with COVID-19. Drug targets among coronaviruses include the main protease 3CL(pro) and papain-like protease(PLpro). These proteinase play essential roles in processing polyproteins and viral replication. The structures of the unliganded SARS-CoV-2 M(pro) and its complex with an alpha-ketoamide inhibitor have been recently revealed [52]. Comparison of genome sequences of SARS-CoV-2 with SARS sequence indicates that the catalytic domains of proteinase are highly conserved in these coronaviruses. Therefore, it is plausible to repurpose the compound library for treatment of SARS-CoV for developing potential therapeutics for SARS-CoV-2. Computational analysis was also used to screen the effective and potent cysteine protease inhibitors for malaria and SARS infection [63,64,65]. Recently, a series of N-(tert-Butyl)-2-(N-arylamido)-2-(pyridin-3-yl) acetamides (ML188) were identified as potent noncovalent small molecule inhibitors targeting SARS-CoV 3CL protease [66,67]. In addition, the analogues of keto-glutamine were developed as potent inhibitors for treatment of SARS infection [68,69]. Furthermore, recent study has shown that compounds containing electrophilic arylketone moiety were designed and synthesized as new SARS-Cov 3CL protease inhibitors [70]. The anilide derived from 2-chloro-4-nitroaniline, l-phenylalanine and 4-(dimethylamino)benzoic acid was found to be a competitive inhibitor of the SARS-CoV 3CL protease with K(i) = 0.03 uM by using a fluorogenic tetradecapeptide substrate [70,71]. It is demonstrated that trioxa-adamantane-triols (TATs) (BN, IBNCA, VANBA, euBN), trivially termed bananins, were identified to be effective inhibitors of SARS-CoV NSP10/nsp13 RNA/DNA helicase/NTPase protein ATPase enzymatic function. Bananin (BN) effectively suppresses both SARS-CoV RNA/DNA helicase nucleic acid unwinding function and SARS-CoV RNA-viral replication in cell culture [72]. In addition, high-throughput screening (HTS) approaches were used to screen potent inhibitors of the SARS-CoV main proteinase [73,74]. Recent computational studies found that lopinavir, oseltamivir and ritonavir are able to bind with SARS-CoV-2 protease [75]. Potential therapeutic options targeting the main protease 3CLpro were identified for SARS-CoV-2, including covalent drugs (approved or clinically tested). There were at least six hits among the total of 11 potential hits identified by using the SCAR protocol [76]. Therefore, it will be intriguing to determine whether these compounds might be effective for inhibiting the activity of proteinase of SARS-CoV-2 in vitro and reducing the replication of the virus in treating novel coronavirus SARS-CoV-2 infection. 

### 5.4. Broad-Spectrum Antiviral Compounds NHC and EIDD-2801

No therapies specific or effective for human coronavirus SARS-CoV-2 have been approved by Food and Drug Administration (FDA). β-D-N4-hydroxycytidine (NHC, EIDD-1931) was initially synthesized as an orally bioavailable ribonucleoside analog with broad-spectrum antiviral activity against various RNA viruses such as Ebola [77]. Recent study discovered that NHC effectively inhibited MERS-CoV and newly emerging SARS-CoV-2 replication using antiviral assays in the human lung epithelial cell line Calu-3 2B4 (“Calu3” cells) [78]. NHC has shown potent antiviral activity with an average half-maximum effective concentration (IC50) of 0.15 μM for cells with a recombinant MERS-CoV expressing nanoluciferase (MERS-nLUC), and there is no observed cytotoxicity. In addition, NHC was potently antiviral with an IC50 of 0.3 μM and CC50 of >10 μM when using a clinically isolated strain of SARS-CoV2 infected African green monkey kidney (Vero) cells [78]. Furthermore, NHC is highly effective for preventing the virus replication of SARS-CoV-2, MERS-CoV as well as SARS-CoV infection in primary human airway epithelial cell cultures [78]. More importantly, NHC inhibited the replication of remdesivir (RDV)-resistant virus and multiple distinct zoonotic CoV [77]. EIDD-2801, an orally bioavailable prodrug of NHC (β-D-N4-hydroxycytidine-5′-isopropyl ester) designed for improved in vivo pharmacokinetics, remarkably reduced SARS-CoV replication and pathogenesis, significantly decreased MERS-CoV infectious titers, and reduced viral RNA and pathogenesis under both prophylactic and early therapeutic conditions in mice [78]. These studies indicate that EIDD-2801 could not only provide effective treatment of SARS-CoV-2 infection, but also enable the prevention of the spread of SARS-CoV-2 and control future outbreaks of other emerging coronaviruses. It is worth noting that animal experiments and human clinical trials are needed to examine its efficacy for treatment of COVID-19.

### 5.5. Application of Anti-Viral Natural Products for Treatment of COVID-19 

Under the current outbreak of COVID-19, it is necessary to repurpose natural products to manage patients with COVID-19. Recent studies have shown that hydroxychloroquine can improve the outcomes of COVID-19 patients in small clinical trials although it should be used with caution on humans due to its toxicity. There are a large number of natural products with known safety profiles, such as isoflavones and artemisinin. Recent study has shown that several isoflavones and related flavonoid compounds have potent antiviral properties. In this regards, natural products that have been repurposed for broad-spectrum anti-viral therapy can offer safe and inexpensive platforms for discovery of efficient and novel agents for treatment of SARS-CoV-2. It will be of significance if these FDA-approved drugs could be repurposed for treatment of COVID-19. 

Recent studies found that naturally occurring flavonoids exhibit a broad-spectrum of antiviral effects against RNA virus such as polio-virus type 1, parainfluenza virus type 3 (Pf-3), and respiratory syncytial virus (RSV) by inhibiting their replication [79]. Computational drug design methods were used to identify Chymotrypsin-like protease inhibitors from FDA approved natural and drug-like compounds [80]. It has been shown that two natural compounds including flavone and coumarine derivatives were identified as promising hits of proteinase inhibitors of SARS-CoV-2 [80]. In addition, recent study has shown that hydroxychloroquine, an anti-malarial drug, significantly abolished SARS-CoV-2 infection [77]. Consistent with this, hydroxychloroquine in combination with azithromycin treatment improved the outcomes of COVID-19 patients in a small clinical study although it should be cautious due to its adverse effects. [81]. Quinine bark was one of the most extensively used therapeutic approached for malaria during the mid-1800s, which provides evidence that chemical compounds from natural products can be used successfully to treat an infectious disease [82,83]. In addition, 36 alkaloids, alcohol extracts and chloroquine are effective in blocking the polymerization process in parasites. One of the main derivatives of quinine, mefloquine, was discovered to suppress the uptake of chloroquine in infected cells by blocking ingestion of hemoglobin to prevent parasite infection [82,83]. Previous study demonstrated that chloroquine inhibited the replication of severe acute respiratory syndrome coronavirus in vitro [84]. Because there is no effective treatment of COVID-19, the extensive outbreak of constant human to human transmission prompts us to apply broad-spectrum anti-viral natural products to prevent or improve the condition of patients with SARS-CoV-2 [43,85]. 

Recent study revealed that the protein sequence of the drug binding pocket of the enzymes is highly homogeneous between SARS-CoV and SARS-CoV-2 [86]. Much progress has been made in the application of natural products and the development of novel therapy for SARS infection [87,88,89,90], for example, a new type of effective inhibitor was identified for inhibition of SARS-CoV proteinase by using substrate specificity profiling [72,91,92]. In addition to chloroquine, an anti-malaria natural product artemisinin has anti-viral activity although the mechanism of artemisinin to in inhibiting virus infection is unknown [93,94,95]. Natural products Tordylium persicum Boiss & Hausskn extract have also been identified for treatment of HIV [96]. Consistent with this, Cuscuta campestris crude extracts have been demonstrated to be effective for inhibition of HIV replication [97]. Cell-based screening assay has been developed to screen virus-specific and broad-spectrum inhibitors for treatment of coronavirus infection [98,99,100]. Therefore, it is necessary to examine the efficacy of artemisinin and other natural products on COVID-19 replication and infection. Another study has shown that the administration of hydroxychloroquine reduced the morbidity of COVID-19 pneumonia [101,102,103]. Chloroquine in clinical trials with a large number of patients will be further examined for treatment of COVID-19 [104]. Some traditional Chinese medicines such as Polygonum cuspidatum that may consist of components with efficacy against COVID-19 have been examined in clinical trials [105]. The pro-inflammatory metabolites of arachidonic acid (AA) and eicosapentaenoic acid (EPA) such as leukotrienes and thromboxanes promote inflammation, whereas lipoxins, resolvins, protectins and maresins derived from AA, EPA and DHA facilitate wound healing, promote phagocytosis of macrophages and other immunocytes and decrease microbial load [106]. It is implicated that these unsaturated fatty acids and pro-inflammatory metabolites may serve as endogenous anti-viral compounds. It is intriguing to determine the efficacy of the metabolites on prevention of SARS-CoV-2 infection [107]. In addition, using structure-based drug selection for identification of SARS-CoV-2 protease inhibitors, old drugs such as macrolides were predicted to be effective against COVID-19 [108]. Therefore, treatments with macrolides alone or in combination with other drugs may be promising and provide the possibility of a new strategy to fight this emerging SARS-CoV-2 infection.

## 6. Spike Glycoproteins of SARS-CoV-2 and ACE2 

**Figure 5:**
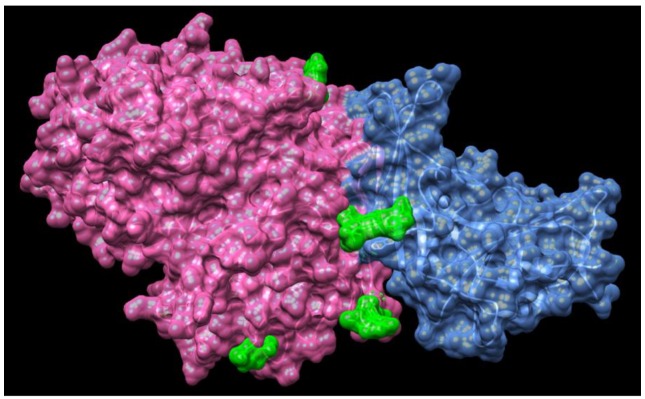
Structures of MERS-CoV spike glycoprotein in complex with sialoside attachment receptors (PDB: 4KR0). MERS-CoV Spike glycoprotein is composed of an N-terminal S1 subunit, which is assembled as four domains (A–D) and controls attachment to dipeptidyl-peptidase 4 (DPP4, the host receptor), and a C-terminal S2 subunit that combines the viral and cellular membranes to initiate infection. Spike glycoprotein is highlighted in pink, DPP4 is highlighted in blue and the ligand Neu5Ac is highlighted in green.

Revealing the structural-functional relationships of the S protein of SARS-CoV-2 will provide new insights into inhibition of interactions between S protein and angiotensin-converting enzyme 2 (ACE2) to develop novel therapeutic approaches for coronavirus. More studies are focused on investigating the mechanism of coronavirus invasion into host cells. Similar to SARS–CoV and MERS-CoV, the novel coronavirus SARS-CoV-2 is armed with a large “spike protein”, which is used to interact with host cells and then gain entry through the cell membrane [109,110]. Recent study has discovered the structure of MERS-CoV spike glycoprotein in complex with sialoside attachment receptors (PDB: 4KR0) [111]. MERS-CoV Spike glycoprotein is composed of an N-terminal S1 subunit, which is assembled as four domains (A–D) and controls attachment to dipeptidyl-peptidase 4 (DPP4, the host receptor), and a C-terminal S2 subunit that combines the viral and cellular membranes to initiate infection, as shown in Figure 5 [111].

**Figure 6:**
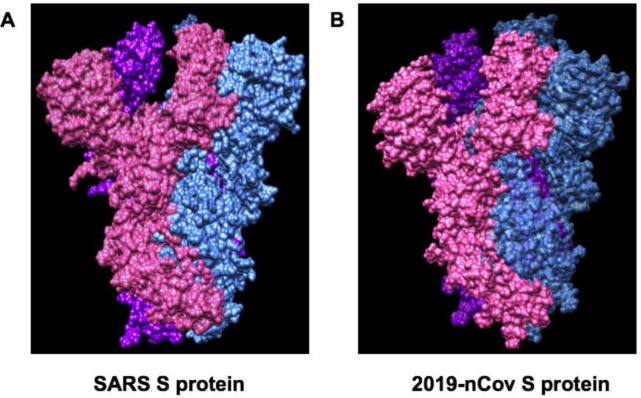
Comparison of Spike glycoprotein structures between SARS (PDB ID:6CRZ) and SARS-Cov-2 (PDB ID:6VSB). (**A**) SARS Spike glycoprotein is composed of NTD, RBD, SD1 and SD2, S2 subunit. The SARS-CoV S trimer is highlighted as a molecular surface with each protomer colored blue, pink or purple. (**B**) SARS-CoV-2 trimer is highlighted as a molecular surface with each protomer colored blue, pink or purple.

Angiotensin-converting enzyme 2 (ACE2) is required for coronavirus invasion into host cells. The viral spike glycoprotein utilizes ACE2 as a host protein receptor and mediates merging of the viral and host membranes. This allows viral entry into host epithelial cells and host species tropism [112]. Given that the structure of S protein (PDB ID:6VSB) of SARS-CoV-2 is revealed [30,86], more computational analysis and virtual screening could be performed to identify the potential inhibitors of S protein and ACE2 interaction as shown in Figure 6. In addition, recent study has shown that S-phase kinase-associated protein 2 (SKP2) is necessary for lysine-48-linked poly-ubiquitination of beclin 1, leading to its proteasomal degradation. Suppression of SKP2 promotes autophagy and decreases MERS coronavirus replication [113]. Recent study demonstrate that SARS-CoV-2 uses the SARS-CoV receptor ACE2 for invasion and the transmembrane protease serine 2 (TMPRSS2) for S protein priming [28]. A clinically proven protease inhibitor, camostat mesylate, has been demonstrated to inhibit Calu-3 infection with SARS-CoV-2 and prevent SARS-2-Spike protein (S protein)-mediated entry into primary human lung cells [28]. In addition, recent study demonstrated that a neutralizing antibody CR3022 targets a highly conserved epitope, distal from the receptor-binding site, that enables cross-reactive binding between SARS-CoV-2 and SARS-CoV. The structure of CR3022 in complex with the receptor-binding domain (RBD) of the SARS-CoV-2 spike (S) protein has been revealed. The modeling study further proved that the binding epitope can only be targeted by CR3022 when the conformational changes with two RBD on the trimeric S protein are in the "up" orientation. This provides a molecular mechanism in the binding of the antibody with S protein of SARS-CoV-2 [114]. In line with this result, recent study demonstrates a highly potent pan-coronavirus fusion inhibitor targeting its spike protein that harbors a high capacity to mediate membrane fusion, and inhibited SARS-CoV-2 infection [115]. The molecular mechanisms of coronavirus invasion into host cells will provide new insights into the development of therapeutic approaches for COVID-19 by targeting spike proteins and ACE2 [116,117,118]. 

## 7. Clinical trials of Remdesivir for Treatment of COVID-19 in China

The outbreak of COVID-19, and previous devastating SARS and MERS-CoV, highlight the importance for developing effective approaches for treatment of human coronavirus infections (SARS coronavirus anti-infectives). This will decrease risk of disease dissemination, ameliorate disease progression, and bring down the need for intensive supportive care. Furthermore, treatments for moderate cases to decrease the time span of illness and infectivity may also be of significance for preventing COVID19 from becoming more wide-spread. Recent study demonstrated that there was no observed benefit with lopinavir–ritonavir treatment in hospitalized adult patients with severe COVID-19 [119]. Future trials in patients with severe illness may help to confirm or exclude the possibility of treatment benefit. We mainly compare two ongoing clinical trials with remdesivir for treatment of SARS-CoV-2 infection. The first one targets mild/moderate patients, the second severe cases with SARS-CoV-2 infection.

### 7.1. Treatment of Mild/Moderate Case of COVID-19 with Remdesivir RCT (ClinicalTrials.gov Identifier: NCT04252664)

**Figure 7:**
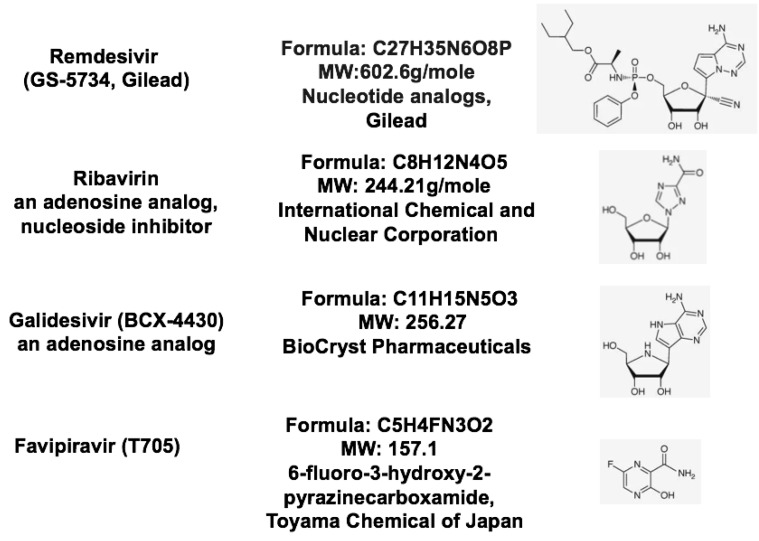
Structures of nucleotide substrate analogues.

Because there is no specific antiviral treatment for COVID-19 infection, the widely investigated small molecule compound remdesvir could be a potential antiviral agent, based on pre-clinical studies in SARS-CoV and MERS-CoV infections. Remdesivir is a 1′-cyano-substituted adenosine nucleotide analogue prodrug that can be metabolized into its active form to exhibit broad-spectrum antiviral activity against coronavirus as shown in Figure 7. A phase 3 randomized, double-blind, placebo-controlled study was designed to examine the efficacy of remdsivir in adult patients with mild/moderate COVID-19 respiratory disease. The inclusion criteria include: (1) laboratory RT-PCR confirmed patients infected with SARS-CoV-2; (2) lung involvement confirmed with CT imaging. The exclusion criteria include: patients with SaO2/SPO2≤94% in room air condition; severe liver disease and severe renal impairment; and patients with any experimental treatment for COVID-19 (off-label, compassionate use, or trial related). 308 participants have been recruited and the clinical trial outcome will be released at the end of April 2020.

### 7.2. Treatment of Severe Case of COVID-19 with Remdesivir RCT (ClinicalTrials.gov Identifier: NCT04257656)

There are no therapeutics proven effective for the treatment of severe illness caused by SARS-CoV-2, so a phase 3 randomized, double-blind, placebo-controlled study was designed to examine the efficacy of remdsivir in adult patients with severe COVID-19 respiratory disease. The clinical trial is sponsored by Capital Medical University and China-Japan Friendship Hospital. The inclusion criteria include: (1) confirmation of COVID-19 by using laboratory RT-PCR; (2) less than 12 days since symptom; (3) lung involvement confirmed with CT chest imaging; (4) patients with a SaO2/SPO2 ≤ 94% in room air condition. The exclusion criteria include: severe liver disease and severe renal impairment; and patients with any experimental treatment for COVID-19 (off-label, compassionate use, or trial related). 452 participants will be recruited and the clinical trial outcome will be released in May 2020. In adults presenting with hypoxic respiratory failure or acute respiratory distress syndrome (ARDS) from COVID-19, invasive mechanical ventilation, a conservative fluid strategy over a liberal fluid strategy, and intermittent boluses of neuromuscular blocking agents (NMBA) to facilitate protective lung ventilation are necessary for supporting treatment [120]. For severe ARDS cases, the routine use of inhaled nitric oxide is recommended [120]. A randomized, controlled clinical trial to evaluate the safety and efficacy of the investigation of antiviral remdesivir in hospitalized patients with COVID-19 has also initiated at the University of Nebraska Medical Center (UNMC) in Omaha. These clinical trials will be of significance for therapy involving severe cases of COVID-19 after completion of examining the efficacy of remdesivir to treat COVID-19. 

Recent studies have shown that SARS-CoV-2 receptor ACE2 and transmembrane protease serine 2 (TMPRSS2) are primarily expressed in bronchial transient secretory cells, which provides the rationale that supporting treatment on lung should be emphasized [121]. The expression of ACE2 can be upregulated by smoking in humans [122], therefore, smoking status should be included for information of identified cases of COVID-19. In addition, it is crucial to manage patients with inherited arrhythmia syndromes such as long QT syndrome and short QT syndrome in the setting of the COVID-19 pandemic [123]. Patients with inherited arrhythmia may be susceptible to pro-arrhythmic factors of COVID-19 such as use of antiviral drugs, fever, stress, and electrolyte imbalance [123,124]. The current studies on potential therapeutic agents, such as lopinavir/ritonavir, favipiravir, chloroquine, hydroxychloroquine, interferon, ribavirin, tocilizumab and sarilumab are important for management of COVID-19 [51,125]. More clinical trials are being conducted for further confirmation of the efficacy and safety of these agents in treating COVID-19 [126].

## 8. Coronavirus and Host Interaction

Several host factors regulate the replication of coronavirus and induce dramatic changes in the host cellular structure and function, simultaneously. Induction of critical signaling proteins is crucial for the pathogenesis of CoV and is also involved in the activation of the innate immune response during CoV infection. Neutrophils are the first immune cells recruited to sites of viral infection. Recent studies demonstrate that excessive recruitment of neutrophils and formation of neutrophil extracellular traps results in acute lung injury of influenza pneumonitis [127]. In addition, neutrophil-derived myeloperoxidase (MPO) serves as a potent tissue damage factor and also contributes to influenza pneumonia in mice infected with influenza virus [128,129,130,131]. In addition, coronavirus infection is able to induce stress response, autophagy, apoptosis, and activate innate immunity [40]. Host cell apoptosis caused by CoV infection has been extensively studied, for example, SARS-CoV and MERS-CoV infect and induce apoptosis in a variety of tissues including lung, spleen, thyroid tissues, and a variety of cell types, such as respiratory epithelial cells, neuronal cells, primary T lymphocytes, and dendritic cells [132,133]. In addition, overexpression of SARS-CoV S was shown to induce a potent ER stress response, and the re-arrangement of DMV production and membrane alteration for the assembly of the virus may contribute to endoplasmic reticulum (ER) stress during CoV infection [40]. Unfolded protein response (UPR) -mediated signaling pathway was induced to maintain ER homeostasis [134,135]. The IRE1 branch of UPR promotes cell survival during CoV infection and CoV infection activates JNK to modulate apoptosis induction [136,137]. Induction of cell apoptosis provided the possible explanations for the fact that lymphopenia was observed in some patients with CoV infection, such as SARS-CoV and SARS-CoV-2. Immune cell apoptosis may lead to the suppression of host immune response and promote the occurring of ARDS.

Coronavirus infection promotes the activation of inflammasome in host cells and activates innate immunity [138,139]. In macrophages, ORF8b provides a potent signal 2 required for activation of inflammasome and activates the NLRP3 (NOD-, LRR- and pyrin domain-containing protein 3) to form the inflammasome complex. Specifically, ORF8b is able to interact directly with the Leucine Rich Repeat (LRR) domain of NLRP3 and bind with apoptosis-associated speck-like protein containing a CARD (ASC) in cytosolic dot-like structures [140]. ORF8b induces cell apoptosis and pyroptotic cell death in macrophages, while in those cells lacking NLRP3, accumulating ORF8b cytosolic substances lead to mitochondrial dysfunction, activation of inflammasome and caspase-independent cell death [140,141]. Recent genome-wide and transcriptome-wide complementary network analysis of SARS-CoV-2/human interaction provided a new network of host proteins affected by the SARS-CoV-2 infection [141]. It has been shown that ACE2 is downregulated in the presence of viral infection, therefore application of recombinant ACE2 has been shown to be effective in treating severe pulmonary infections and acute respiratory distress syndrome [142]. Recent studies demonstrate that soluble forms of ACE2 are beneficial to SARS patients, due to its competitive binders of SARS-CoV Spike proteins, preventing binding to the host cell ACE2 [143].

## 9. Concluding Remarks

Recent study indicate that there is a substantial number of undocumented infections and this facilitates the rapid dissemination of SARS-CoV2 [144]. These findings explain the rapid geographic spread of SARS-CoV2 and indicate containment of this virus will be particularly challenging. It is also informative to help understand the potential for infection by non-symptomatic subjects. Therefore, one of the most important strategies of management of COVID-19 infection is to effectively reduce the possibility of constant human-to-human viral transmission [145]. Keeping social distancing, contact tracing and quarantine are necessary for preventing substantial human to human transmission of COVID-19 [146,147,148]. Recent study demonstrated that the affinity of the SARS-CoV-2 S protein with ACE2 was 10- to 20-fold higher than the SARS-CoV S protein as shown in Figure 5 [30], which explains the observations of rapid transmission from human-to-human in COVID-19 infection. Genetic analyses of hundreds of SARS-CoV-2 genomes revealed that there were two major types, L type and S type [149]. It seems that the L type (∼70%) was more dominant than the S type (∼30%). The L type was more common in the early stages of the outbreak in China. Artificial intervention may have put selective pressure on the L type, which might be more susceptible to mutate and spread more widely [149]. It is very challenging to makes an effective vaccine because of the rapid mutation of ssRNA and the antibody-dependent enhancement (ADE) effect [149]. Therefore, it is urgent to develop effective medicine for treatment of moderate and severe patients with low SpO2. Remdsivir may be potentially developed for treatment of COVID-19 after completing the phase 3 randomized, double-blind, placebo-controlled study to examine its efficacy in patients with COVID-19. In addition, better understanding of the mechanisms of coronavirus invasion into host cells could accelerate discovery of new inhibitors of interaction of spike glycoproteins and ACE2 and promote the development of therapeutic approaches for COVID-19. Finally, by drawing lessons from mechanisms of replication of SARS-CoV-2 RNA-dependent RNA polymerase, development of potent and effective RdRp inhibitors will provide new insights required for putting RdRp targeted therapeutics into full gear. 

## Figures and Tables

**Table 1:** Potential pharmacological therapeutics for COVID-19.

Therapeutic Targets	Functions	Potential Drug Candidates	References
RNA-dependent RNA polymerase, RdRp	An RNA-dependent RNA polymerase for replicating coronavirus genome	Remdesivir, Ribavirin and Favipiravir can inhibit viral RdRp	[24]
Papain-like protease, PLpro	A protease for the conversion of viral polyprotein into functional enzyme	Lopinavir, protease inhibitors that may inhibit the viral proteases: 3CLpro or Plpro. Darunavir inhibits the proteolysis activity of 3-chymotrypsin-like protease.	[50]
Main protease 3CLproteinase, 3CLpro	A protease for the conversion of viral polyprotein into functional protein	Lopinavir, protease inhibitors that may inhibit the viral proteases: 3CLpro or Plpro.	[51,52]
S protein and TMPRSS2	A viral surface protein for binding to host cell receptor ACE2. TMPRSS2, a host cell-produced protease that primes S protein to facilitate its binding to ACE2	Arbidol can prevent S protein/ACE2 interaction and inhibit membrane fusion of the viral envelope by preventing the binding of viral envelope protein to host cells and preventing viral entry to the target cell. Camostat mesylate inhibits TMPRSS2 and viral cell entry	[28,53]
ACE2	A viral receptor protein on the host cells which binds to viral S protein	Chloroquine and hydroxychloroquine can inhibits vial entry and endocytosis by increasing endosomal pH, interfere with ACE2 glycosylation as well as host immunomodulatory effects	[54,55]

## Abbreviations

ARDS	Acute respiratory distress syndrome
ADE	Antibody-dependent enhancement
SARS-Cov-2	Severe acute respiratory syndrome coronavirus 2
COVID-19	Novel Coronavirus disease-2019
3CLpro	Coronavirus main proteinase
ACE2	Angiotensin-converting Enzyme 2
CRISPR	Clustered Regularly Interspaced Short Palindromic Repeats
Cov	Coronavirus
dsRNA	Double strand RNA
DPP4	Dipeptidyl-peptidase 4
ERGIC	ER-Golgi intermediate compartment
EBOV	Ebola virus
gRNA	Guide RNA
GSK3	Glycogen synthase kinase 3
hM-RNAP	Human mitochondrial RNA polymerase
Orf1	Open reading frame 1
PLpro	Papain-like protease
MERS	Middle East Respiratory Syndrome
MPO	Myeloperoxidase
NTD	N-terminal domain
NMBA	Neuromuscular blocking agents
NHC	β-D-N4-hydroxycytidine
N protein	Coronavirus nucleoproteins
NLRP3	NOD-, LRR- and pyrin domain-containing protein 3
RBD	Receptor-binding domain
RT-PCR	Reverse transcription PCR
SARS	Severe Acute Respiratory Syndrome
S protein	Spike glycoprotein
ssRNA	Single strand RNA
SPR	Surface plasmon resonance
SKP2	S-phase kinase-associated protein 2
SpO2	Peripheral capillary oxygen saturation
RdRp	RNA-dependent RNA Polymerase
RBD	Receptor-binding domain
TMPSS2	Transmembrane protease serine 2
TATs	Trioxa-adamantane-triols
UPR	Unfolded protein response
VLPs	Virus-like particles
